# Changing incentives to ACCELERATE drug development for paediatric cancer

**DOI:** 10.1002/cam4.5627

**Published:** 2023-01-16

**Authors:** Teresa de Rojas, Pamela Kearns, Patricia Blanc, Jeffrey Skolnik, Elizabeth Fox, Leona Knox, Raphael Rousseau, François Doz, Nick Bird, Andrew J. Pearson, Gilles Vassal

**Affiliations:** ^1^ ACCELERATE Brussels Belgium; ^2^ Cancer Research UK Clinical Trials Unit National Institute for Health Research (NIHR) Birmingham Biomedical Research Centre, Institute of Cancer and Genomic Sciences Birmingham UK; ^3^ Imagine for Margo ‐ Children Without Cancer Saint‐Germain‐en‐Laye France; ^4^ INOVIO Pharmaceuticals, Inc. Plymouth Meeting Pennsylvania USA; ^5^ St Jude Children's Research Hospital Memphis Tennessee USA; ^6^ Solving Kids' Cancer UK London UK; ^7^ Gritstone Oncology, Inc. Emeryville California USA; ^8^ SIREDO Centre (Care, Innovation Research in Paediatric, Adolescent and Young Adult Oncology) Institut Curie Paris France; ^9^ Université Paris Cité Paris France; ^10^ Paediatric and Adolescent Oncology Department, Gustave Roussy Cancer Campus, INSERM U1015 Université Paris‐Saclay Villejuif France

**Keywords:** drug development, incentives, paediatric oncology, paediatric regulation, supplementary protection certificate

## Abstract

**Background:**

More effective incentives are needed to motivate paediatric oncology drug development, uncoupling it from dependency on adult drug development. Although the current European and North‐American legislations aim to promote drug development for paediatrics and rare diseases, children and adolescents with cancer have not benefited as expected from these initiatives and cancer remains the first cause of death by disease in children older than one. Drug development for childhood cancer remains dependent on adult cancer indications and their potential market. The balance between the investment needed to execute a Paediatric Investigation Plan (PIP) in Europe and an initial Paediatric Study Plan (iPSP) in the US, coupled with the potential financial reward has not been sufficiently attractive to incite the pharmaceutical industry to develop drugs for rare indications such as childhood cancer.

**Methods:**

We propose changes in the timing and nature of the rewards within the European Paediatric Medicine Regulation (PMR) and Regulation on Orphan Medicinal Products (both currently under review), which would drive earlier initiation of paediatric oncology studies and provide incentives for drug development specifically for childhood indications.

**Results:**

We suggest modifying the PMR to ensure mechanism‐of‐action driven mandatory PIP and reorganization of incentives to a stepwise and incremental approach. Interim and final deliverables should be defined within a PIP or iPSP, each attracting a reward on completion. A crucial change would be the introduction of the interim deliverable requiring production of paediatric data that inform the go/no‐go decisions on whether to take a drug forward to paediatric efficacy trials.

**Conclusion:**

Additionally, to address the critical gap in the current framework where there is a complete lack of incentives to promote paediatric‐specific cancer drug development, we propose the introduction of early rewards in the Orphan Regulation, with a variant on the US‐Creating Hope Act and its priority review vouchers.

## Graphical abstract

More effective incentives are needed to motivate paediatric oncology drug development, uncoupling it from dependency on adult drug development. We propose substantial changes in the timing and nature of the rewards within the European Paediatric Medicine Regulation (PMR) and Regulation on Orphan Medicinal Products (both currently under review), which would drive earlier initiation of paediatric oncology studies and provide incentives for drug development specifically for childhood indications. Incentives should be implemented earlier rather than later in the drug development process, and be staged, milestone‐driven, novel, proportional to work completed at each phase and transferrable.

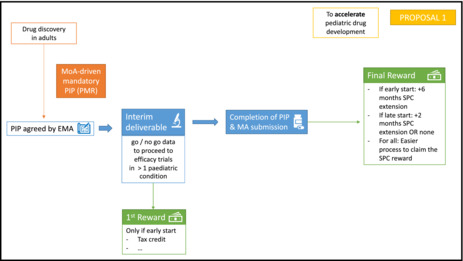

## 1 INTRODUCTION

Over 400,000 children and adolescents are diagnosed with cancer globally each year, 50,000 in Europe and North America.^1^, ^2^, ^3^, ^4^ While there has been improvement in survival since the 1970s, the decrease in mortality has reached a plateau—in high‐income countries, approximately 20% of patients will die of their disease or of disease‐related causes; paediatric cancer remains the first non‐accidental cause of death in children and adolescents.^4^, ^5^ Therefore, there is an urgent need for new medicines to cure aggressive tumours, and to reduce the toxicity and sequelae of the treatments.^6^ Evaluating new anti‐cancer drugs in paediatric patients is critical ethically and enrolment in early‐phase clinical trials is an option to be proposed to the patient and family.^7^ Children with relapsed/refractory disease ethically deserve the option of a clinical trial when no curative treatment is known. Moreover, these trials need to be scientifically robust and of the highest quality.

The European Regulation on Orphan Medicinal Products for medicines for rare diseases and the Paediatric Medicines Regulation (PMR) were adopted in 2001 and 2007, respectively, aiming to improve treatment options for these patients.^8^, ^9^, ^10^ At the time, limited or no relevant data on medicinal products were available for either group (patients with rare disease and paediatric patients)—both to which children and adolescents with cancer belong. The market size was mostly small, and developing medicines and conducting clinical trials was more complex.^10^ A combination of obligations, incentives and rewards was introduced with both regulations, to address the apparent market failure.^10^ The objectives of the two regulations partly overlap, as many diseases that affect only children are rare and rare diseases often also affect children, as is the case of paediatric cancers.^10^

In 2016, the European Parliament recognized that the PMR had been beneficial to children overall, but not sufficiently effective in certain therapeutic areas—notably paediatric oncology^10^, ^11^—and called on the Commission to revise the Regulation. The revision of the two legislations is also one of the actions of the European Union (EU) Pharmaceutical Strategy.^10^, ^12^ The evaluation carried out in 2020 by the Commission^13^ showed that both legislative instruments have stimulated research and development of medicines to treat rare diseases and of medicines for children.^10^ However, it also showed shortcomings in the functioning of the existing legal framework. This is partly due to the legislation not being able to stimulate development of medicines in areas of unmet needs, such as childhood cancers and neonatology, that is a failure of the existing incentives.^10^, ^14^

In the United States (US), the Best Pharmaceuticals for Children (BPCA),^15^ Paediatric Research Equity Act (PREA),^16^ and the Research to Accelerate Cures and Equity Act (RACE)^17^ as well as Rare and Orphan Drug Designations aim to encourage those developing drugs to implement paediatric cancer programmes early on in development; however, limited success has been achieved so far. The objective of the Creating Hope Act was to incentivise sponsors to develop new medicines specifically for children suffering of life‐threatening diseases, rewarding such efforts with Priority Review Vouchers (PRV), which reduce the FDA's review time of a specific product from ten to six months.

Between 2008 and 2022, with both major EU and US legislations in place (the PMR since 2007^9^ and the PREA since 2003,^16^ respectively), only 29 anti‐cancer new molecular medicines were approved with a paediatric indication (16 in the EU, 29 in the US).^18^ Conversely, 133 anti‐cancer medicines were approved for adults in the EU in that same period. Furthermore, the paediatric development of many potentially relevant anti‐cancer drugs for children has been waivered on the ground that the condition for which they are indicated in adults does not occur in children (for example, lung cancer). There is an urgent need to examine why the European and North‐American legislation has fallen short of expectations in this disease area and to consider potential actions that will ensure children and adolescents with cancer can derive the intended benefits.

ACCELERATE, an international paediatric oncology platform involving multiple stakeholders (academia, industry, regulatory bodies and patients and families) was established to hasten paediatric oncology drug development within the current regulatory framework.^5^, ^19^ A Working Group of ACCELERATE was convened to propose more effective incentives for paediatric‐specific oncology drug development.^7^ This Working Group's conclusions are very timely in view of the ongoing revision of the European PMR and Orphan regulations. This article outlines the current framework of incentives for paediatric drug development, highlights the lack of impact of the current incentives framework on childhood cancers and proposes changes to the current EU and US legislative framework.

## 2 CURRENT FRAMEWORK OF INCENTIVES FOR PAEDIATRIC DRUG DEVELOPMENT

The main European and North‐American legislation (PMR; Regulation on Orphan Medicinal Products; US Creating Hope Act; and RACE Act) are summarized in Table 1. Further details are described in the Appendix S1.

**TABLE 1:** Current legal framework of obligations and incentives in paediatric drug development

Site	Law	Obligations	Waiver	Incentives	Timelines/conditions
EU	Paediatric Medicines Regulation (EC No 1901/2006)	When developing a drug for MA, submit a PIP, including: Studies in applicable age groups (0–17 years) Drug formulation adaptations	Medicine is likely to be either ineffective or unsafe does not have substantial therapeutic benefit over existing treatments is intended to treat a condition that only occurs in adults	6‐month market exclusivity extension if the PIP is completed as agreed For drugs with orphan drug designation: additional 2‐year extension (i.e. total orphan market exclusivity increases to 12 years)	PIP must be submitted after completion of adult PK studies (i.e. end of phase 1 trials)
	Medicines for Rare Diseases Regulation	NA	NA	Reduction in fees for MA applications 10‐year orphan market exclusivity	Drug must be intended to treat, prevent or diagnose a disease that is life‐threatening or chronically debilitating Disease prevalence <5 in 10,000 or the product's market unlikely to generate sufficient returns to justify the investment Significant benefit to patients from the new treatment or no satisfactory method of treatment in the EU
USA	Best Pharmaceuticals for Children Act	NA	NA	Additional 6 months of marketing exclusivity to sponsors who voluntarily complete paediatric clinical studies as outlined by a Written Request (WR) issued by FDA Sponsors can also request a WR for drugs under development	Sponsors must adhere to the WR and perform studies in line with the FDA WR
	Creating Hope Act (2012)	NA	NA	Priority Review Voucher: reduces FDA review time from 10 months to 6 months, gaining 4 months of market access. Transferable: for a different drug and indication (with a broader marketing potential) and can be sold to another company	If a company obtains market approval for a drug for a life‐threatening/severe paediatric indication (including cancer)
	PREA	FDA may require paediatric studies in certain drugs and biological products and requires sponsors to create PSP to define their paediatric drug development strategy. It also requires the use or creation of age‐appropriate formulations.	Orphan drug designation Disease occurs only in adults	NA	PSP to be submitted after the (adult) end‐of‐phase 2 meeting
	RACE Act (2017, effect in 2020)	Requires paediatric evaluation (submission of PSP) of new molecularly targeted drugs and biologics intended for the treatment of adult cancers and directed at a molecular target substantially relevant to the growth or progression of a paediatric cancer.	NO longer waiver because (as opposed to PREA): Orphan drug designation Disease occurs only in adults Mechanism of action/target is considered now	NA	PSP to be submitted after the (adult) end‐of‐phase 2 meeting

Abbreviations: MA, Marketing authorization; NA, Not Applicable; PIP, Paediatric Investigation Plan; PK, pharmacokinetic; PREA, Paediatric Research Equity Act; PSP, Paediatric Study Plan; WR, Written Request.

**FIGURE 1:**
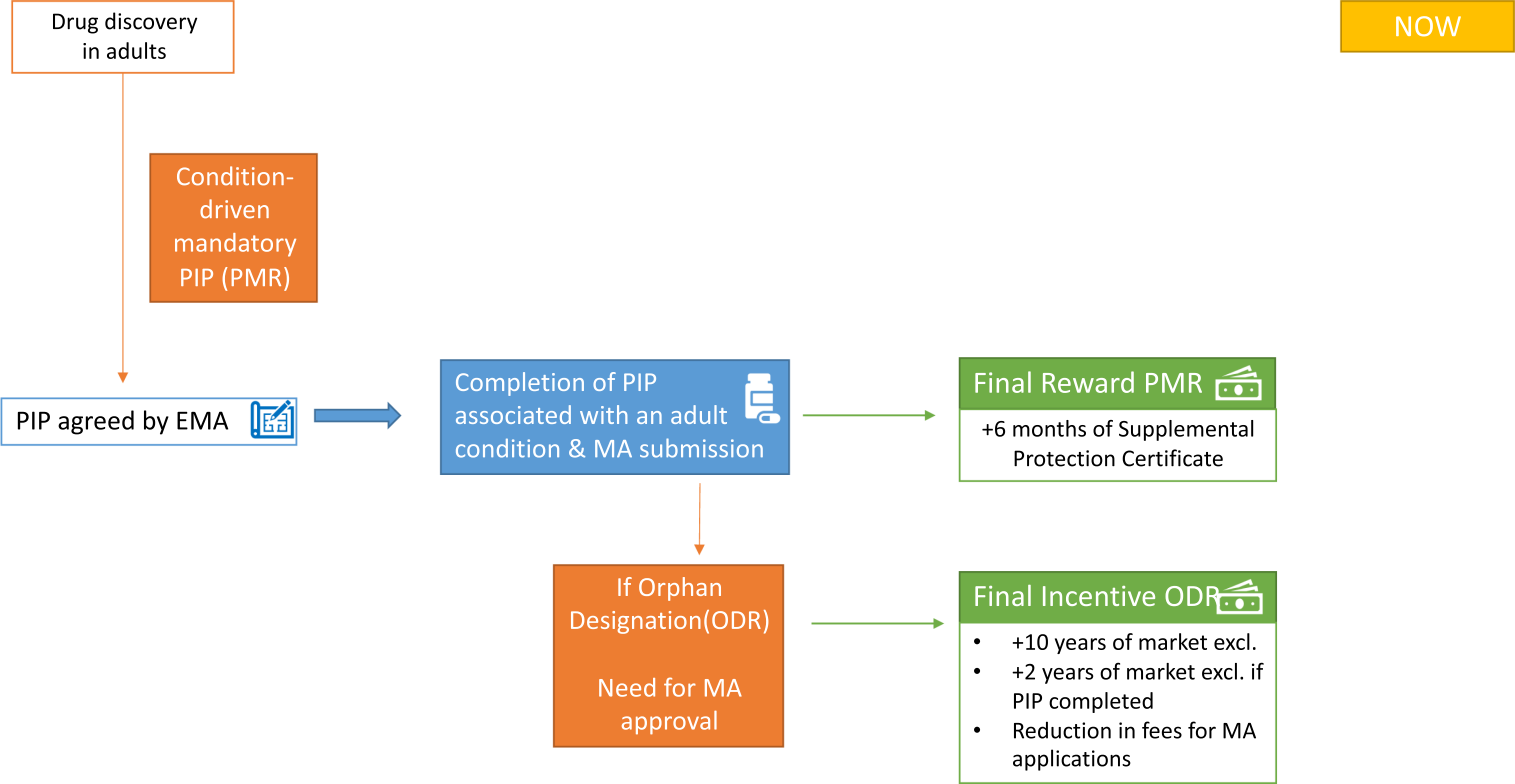
Current legal framework of incentives for paediatric drug development in the EU. Under the Paediatric Medicines Regulation, paediatric investigation plans (PIPs) are condition‐driven and follow drug discovery in adults. A reward can be obtained on delivery of a completed PIP and updated product labelling. Additional incentives can be achieved if the drug receives orphan designation. EMA, European Medicines Agency; MA, Marketing Authorization; ODR, Orphan Drug Regulation; PIP, Paediatric Investigation Plan; PMR, Paediatric Medicines Regulation

The current EU framework to promote drug development for children and adolescents includes both a regulatory obligation (with the possibility of waivers if the condition for which the product is intended does not occur in the paediatric age group, if the drug is likely to be either ineffective or unsafe, or if it does not have substantial therapeutic benefit over existing treatments) and the financial rewards (6 months of extended Supplementary Protection Certificate (SPC), i.e. of market exclusivity) on delivery of a completed Paediatric Investigation Plan (PIP) and updated product labelling, regardless of the results of the paediatric trials. It is, therefore, not dependent on the PIP demonstrating benefit of the drug in a paediatric population (Figure 1). According to the EU legislation, companies can file for a new marketing authorization in adults when they have an agreed PIP or a waiver. In practice, this implies that companies can submit for marketing authorization or applications for variation of an existing one, as soon as the PIP is approved because a deferral of the start of the PIP can be granted, again delaying paediatric evaluation.

In the US, the FDA developed the BPCA to create the incentive of additional marketing exclusivity to sponsors who voluntarily complete paediatric clinical studies as outlined by a Written Request issued by FDA.^20^ Sponsors can also request a Written Request for drugs under development. Meeting the requirements of the request grants sponsors six additional months of market exclusivity, but sponsors must adhere and perform studies in line with the FDA Written Request.

The RACE for Children Act, which took effect in 2020, requires paediatric evaluation (submission of an initial Paediatric Study Plan, iPSP) of new molecularly targeted drugs and biologics intended for the treatment of adult cancers and directed at a molecular target substantially relevant to the growth or progression of a paediatric cancer.^17^ The drug evaluation is made on a mechanism‐of‐action approach, and therefore, waivers cannot be obtained on the grounds of the disease only occurring in adults.

The Creating Hope Act, enacted in 2012 and reauthorized by the US Congress in 2020 for an additional 4 years, aims to provide PRVs to those sponsors who voluntarily prioritized paediatric drug development by labelling a drug to treat a rare paediatric disease.^21^ ‘Rare’ is defined pursuant to the Orphan Drug Act, that is it affects fewer than 200,000 Americans; ‘paediatric’, pursuant to the FDA Guidance for Industry, that is over 50% of the patients present with the disease before age 18. The disease itself must also qualify for priority review—it must be life‐threatening and address an unmet medical need. The voucher entitles the marketing authorization holder to the priority review of another single human drug or biologics application, which has the potential to provide an economic and competitive advantage to medicines vying for first‐to‐market status by decreasing time to approval. Because the voucher is transferable, the recipient can sell it to another company.

## 3 LACK OF IMPACT OF THE CURRENT INCENTIVES FRAMEWORK ON CHILDHOOD CANCERS

Whilst the regulatory imperatives have led the pharmaceutical industry to actively consider patients younger than 18 years of age in their drug development programmes, the balance between the level of investment needed to execute a PIP and the potential financial reward is not proving sufficiently attractive for rare indications such as childhood cancer; a waiver or deferral of the PIP being the common outcome.^22^ This was shown by a 2016 study on the economic impact of the PMR, which concluded that whilst the regulation is a commendable first step, there remain therapeutic areas where significant unmet needs continue to exist, such as in childhood cancer.^23^ This study estimated the total cost of the PMR incurred to industry to be €2106 m per year or €16,848 m for the years 2008–2015.^23^ It also analysed the economic value of the rewards provided under the PMR, by analysing the SPC extensions covering eight medicinal products, which received SPC extensions in the period between 2007–2012 and lost their exclusivity before the third quarter of 2014.^23^ The economic value as a percentage of 6‐month revenue varies between 11% and 94%.^23^ The combined economic value (or monopoly rent) of the eight products is calculated to amount to €517 m, with an extrapolated economic value of €926 m between 2007–2015.^23^ Therefore, the authors believe that ‘the objectives of the reward scheme are deemed highly relevant when considering that the rewards provide a way for organizations to sponsor and support the development of paediatric medicines. Nevertheless, the rewards themselves cannot guarantee capital allocation decisions that maximize value for companies or result in positive return on investment in individual Research and Development programmes’. Current incentives facilitate pharmaceutical companies to invest in the development of paediatric drugs, but they do not guarantee this investment will lead to the economic return that companies plan or hope for.

In oncology, this means that industry investment in paediatric cancer trials is usually either absent or delayed and cancer drug development programmes remain inextricably linked to the market potential for adult cancer indications.^7^ Nader et al. have recently shown that the median times from first‐in‐adult to first‐in‐paediatric for monotherapy and combination trials are 5.7 and 3.3 years, respectively.^24^ This supports our contention that there is inadequate motivation for the pharmaceutical industry to focus on cancer drug development for paediatric cancer‐specific markets with no adult cancer marketing value.

Vassal et al. have demonstrated that between 1995 and 2022, 186 medicines received a first marketing authorization for the treatment of cancer in Europe—however, only 29 had a paediatric indication.^18^ Most of these (23/29, 79.3%) were approved after the implementation of the PMR (2008–2022). Out of the 23 drugs approved since 2007 with a paediatric indication, most were studied within a PIP (18/23, 78%). The first drug to be approved as part of a PIP was everolimus in 2011 for the treatment of subependymal giant cell astrocytoma.

Therefore, two main challenges are identified: First, to drive earlier initiation of paediatric studies according to a mechanism‐of‐action driven decision following drug discovery in adults, and second, to provide incentives for drug development specifically directed at cancers occurring only in children (paediatric‐specific drug development).

The industry's perspective to not pursue research and development in areas that will not be commercially viable is understandable in business terms. Therefore, if the current incentives are inadequate for a company to see any economic advantage to continue paediatric cancer drug development of a medicinal product when the medicinal product is paediatric‐specific, what is needed for industry to be motivated to continue such development for rare indications in the absence of an associated lucrative commercial market?

There are four scenarios for cancer drug development since the PMR was implemented, which are portrayed in Table 2; scenario 3 will not be discussed in this manuscript.

**TABLE 2:** Scenarios for cancer drug development since the implementation of the Paediatric Medicine Regulation in the EU

#	Adults	Children	Examples	Compliance with PMR
1	Drug developed and marketed	**Subsequently** developed for the paediatric. population, BUT: same indication as adults	Delayed development of BCR/ABL‐inhibitors for Ph + chronic myeloid leukaemia ➔ MA for dasatinib in adults in 2006 (USA and EU), for children in 2017 (USA) and 2018 (EU) Waiver for crizotinib on the grounds that lung cancer does not occur in children	Not in compliance. If the disease occurs both in adults and children, paediatric development should not be delayed (nor waivered)
2	Drug developed and marketed	Developed in **parallel** in children with a different indication ➔ based on mechanism of action, not on disease indication	Parallel development of BRAF inhibitors for melanoma and lung cancer in adults, AND for BRAF‐mutated brain tumours and histiocytosis in children Agreed PIP for the combination of dabrafenib + trametinib for glioma with BRAF‐V600 mutations (1–18‐year‐old patients)	In compliance. PIP should be submitted not later than after completing PK studies in adults, if PMR is implemented BUT: the possibility of deferral on the initiation of the PIP studies results often in delays
3	Drug developed but NOT marketed	Development **stopped** even though scientific rationale for developing drug in paediatric population	Development of IGF1‐R antibodies was stopped due to failure for adult indications (lung, breast, pancreatic cancer) ➔ despite compelling pre‐clinical and early‐phase clinical evidence of potential benefit in children, especially in Ewing sarcoma	This is in compliance with PMR and a result of lack of (economic) incentives for companies to continue paediatric development if there is no market benefit in adults
4	No	Drug developed and marketed **first**‐in‐paediatric. population ➔First‐in‐child MA	Dinutuximab (MA in 2015 in EU and USA), first‐in‐class anti‐GD2 antibody for neuroblastoma	Not driven by the PMR. The initial effort was done by academia, followed by industry‐led development, motivated by US incentives The company benefited from the US Creating Hope Act ➔ Transferable Priority Review Voucher

Abbreviations: IGF1‐R, anti‐Insulin‐like growth factor 1 Receptor; MA, Marketing authorization; NA, Not Applicable; PD‐1, Programmed cell death protein; PIP, Paediatric Investigation Plan; PK, pharmacokinetic; PMR, Paediatric Medicine Regulation.

In the USA, while BPCA encourages paediatric drug development, and hundreds of written requests have been submitted, few have resulted in quantifiable changes in drug development for children with cancer, and rewards have been limited, resulting in only 17 paediatric label changes across the entire 20‐year history of the programme as of February 2022.^25^

The Creating Hope Act enabled the use of PRVs and was seen as a very positive step. In practice, however, only three PRVs have been awarded for a paediatric cancer: dinutuximab and naxitamab for neuroblastoma and tisagenlecleucel for B cell acute lymphoblastic leukaemia. In part, this is because in order to obtain a voucher, the product must be approved, and approved first, in a paediatric indication for a disease where a rare paediatric disease designation has been granted for the product; supplementary approvals following adult indications do not qualify for voucher reward (the converse is not true; once approved in paediatrics, the drug may be developed in adult diseases as well). It is also noteworthy that vouchers are non‐discriminatory in terms of potential market size, that is percentage of population. Frontline or narrow relapse/refractory disease indications afford the same reward.

The 2020 study released by the US Government Accountability Office (GAO) investigated the effectiveness and overall impact of the PRV programme.^26^ Between 2009 and 2019, 31 PRVs were awarded, mostly for drugs to treat rare paediatric diseases, out of which 17 were sold to another drug sponsor for prices ranging from $67 million to $350 million.^26^ In this report, GAO found few studies that examined the PRV programmes, and those that did found the programmes had little or no effect on drug development.^26^ However, the participating drug sponsors stated that PRVs were a factor in drug development decisions.^26^ Some academic researchers and stakeholders expressed concerns about the PRVs as incentives for drug development.^26^, ^27^, ^28^, ^29^, ^30^ As defended by Meyer, the GAO report ‘shows weak evidence of PRVs truly incentivizing development’.^29^ This author recommends that critical appraisals ‘must include how drug development and regulatory review have changed since 2007, as well as experience with drug pricing of products granted PRVs’.^29^ Other authors, like Hwang et al., find the impact of the PRV to be more positive, yet still recommend changes.^30^ They concluded that the voucher programme was not associated with a change in the rate of new paediatric drugs starting or completing clinical testing, but there was a significant increase in the rate of progress from Phase I to Phase II clinical trials after the programme was implemented.^30^ Hence, new policies may be needed to expand the pipeline of therapies for rare paediatric diseases.^30^

## 4 REVISING INCENTIVES TO ACCELERATE PAEDIATRIC CANCER DRUG DEVELOPMENT

**FIGURE 2:**
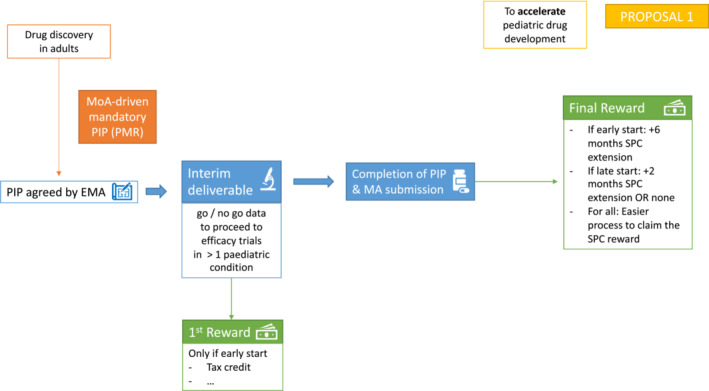
Proposal to accelerate paediatric drug development. Proposal 1 aims at driving earlier initiation of paediatric studies in a mechanism‐of‐action driven environment. Paediatric investigation plans (PIPs) should be considered as a more iterative process with defined interim and final deliverables, each attracting rewards in their own right (first and final rewards). EMA, European Medicines Agency; MA, Marketing Authorization; PIP, Paediatric Investigation Plan; PMR, Paediatric Medicines Regulation; SPC, Supplemental Protection Certificate

**FIGURE 3:**
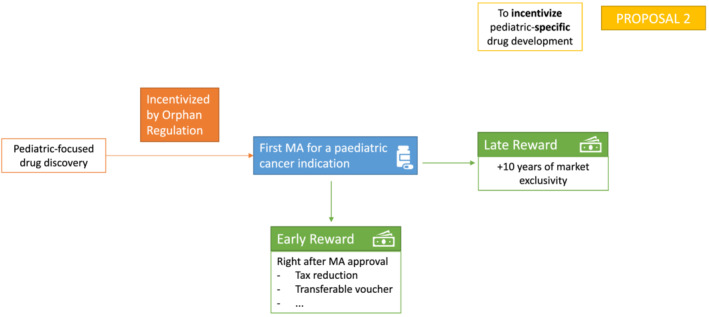
Proposal to incentivise paediatric‐specific drug development. Proposal 2 aims at providing incentives for drug development specifically directed at cancers occurring only in children. A new early reward would be granted right after a first marketing authorization for a paediatric cancer indication, warranting an immediate economic gain, as opposed to the classic late reward. This late reward would, however, remain as it is now. MA, Marketing Authorization

The PMR has not accelerated paediatric and adolescent cancer drug development, to the degree needed; therefore, the pivotal question is how can a better reward or incentive framework accelerate development? Whilst the PMR has successfully motivated the pharmaceutical industry to focus on paediatric drug development for many paediatric diseases, we need to consider if a revision in the incentives framework could extend the benefit to children and adolescents with cancer. As discussed above, two challenges emerge that we have translated into proposals 1 and 2: To accelerate paediatric drug development in a mechanism‐of‐action driven environment (Figure 2) and to provide incentives for drug development specifically directed at cancers occurring only in children (Figure 3).

### 4.1 Proposal 1: drive earlier initiation of paediatric studies (accelerate paediatric drug development)

If a drug does have a potential adult market, and there is also a potential application of the drug in a paediatric condition not relevant to the adult market, it should be viable for the two development programmes to proceed in parallel, neither depending on the success of the other. This would be the case where a drug's mechanism of action is relevant to different cancers in the adult and paediatric age groups (scenario #2).^31^ The RACE legislation ensures this in the United States, and we propose that in Europe, the PMR should be modified to ensure a mandatory mechanism‐of‐action driven PIP. This is the first necessary modification to enhance paediatric drug development. To further encourage and accelerate development, incentives need to be introduced at an earlier stage in the clinical development pathway, rather than only at the end of the SPC, with a staged and milestone‐driven approach.

Currently, it is mandated by the PMR that the package of proposed studies that constitute a PIP are submitted not later than upon completion of human pharmacokinetic studies in adults (i.e. completion of first phase 1 trials for oncology products) and should include the study synopses for all the planned studies of paediatric relevance that would be required for the medicinal product's application for marketing authorization in the paediatric condition. This should include non‐clinical studies, pharmacokinetic and dose finding studies, as well as phase III efficacy studies that often require specifics about a proposed comparator or a randomized design. Whilst all this is obligatory, the reward for this investment is not realized until the PIP is completed and all conditions of the PMR are met. Therefore, the delivery of PIPs for rare paediatric cancers can be challenging and there are several stages at which the full PIP could fail to be delivered, negating the potential reward despite the up‐front investment.

We propose that PIPs should be considered as a more iterative process with defined interim and final deliverables, each attracting rewards in their own right (Figure 2). The aim would be to encourage industry to initiate the PIP earlier in the drug's development pathway with the potential for an earlier reward based on key go/no‐go milestones. This could reduce the current tendency for deferral of initiation of all the paediatric studies until completion of the adult development. For example, the completion of the first clinical studies described in the PIP up to and including the early‐phase clinical trials would provide crucial data on the age‐relevant safety profile (including infants and younger patients where relevant and feasible), pharmacokinetics, pharmacodynamic endpoints and potentially an activity signal for the drug. These data could inform a go/no‐go decision on further development in the paediatric age group and could, therefore, be defined as the first deliverable within a given timeframe within the PIP. This first reward would hence only be given if paediatric development is started early and could consist of tax credit or other forms of economic gain that do not depend on eventual market authorization and exclusivity. It would be important to include a mandate to complete the PIP, with an incentive, if a positive ‘go signal’ is met at the first milestone.

Subsequent efficacy studies would constitute a second/final deliverable, again within a given timeframe. This would lead to a final reward on submission for marketing authorization that could again be stratified. If paediatric development was started early during the process, additional 6 months of market exclusivity could be granted. If, however, paediatric studies were delayed, the extension of market exclusivity could be reduced to 2 months (or to no extension at all). In all instances, the processes to benefit from SPC rewards should be made easier. The issues with the current SPC legislation have been recognized by the Commission, which published an evaluation in 2020 that concluded that the main shortcoming is the fact that SPCs are granted and administered nationally.^32^ SPC applications are currently filed at the national patent office of each EU Member State where protection is sought and in which a basic patent, to be extended, has been granted. This undermines their effectiveness and efficiency, leading to high cost and administrative burden for the SPC users, among other issues. The legislative proposal to address these issues are planned to be adopted by the end of 2022.^33^

If the PIP deliverables are segmented, then the rewards described in the PMR can be proportionately awarded if the deliverables are achieved in the prescribed timeframe. The introduction of this ‘segmented reward approach’ for completion of an interim deliverable would be a significant change to the current PMR and could facilitate a more iterative approach to the design of studies within the PIP, with efficacy studies being informed by the preceding early‐phase studies and the contemporaneous clinical trials landscape in the diseases being studied (Figure 1).

The proposed change to the submission requirements of PIPs would embed reward for early initiation of paediatric studies. A company would be encouraged to submit their proposal for phase 1 paediatric study plans before commencement of the adult phase 2 trial—this would be reflected in the reward but would not be mandatory. This phase 1 study element of the PIP could include age‐relevant data on safety, pharmacokinetic and (where relevant) pharmacodynamic endpoints in at least one paediatric condition. Based on the results of the phase I trial and the predefined go/no‐go decision, the company would be required to submit the plans for the phase II and phase III study elements of the PIP before it is able to submit its application for the marketing authorization on the adult indication. This would be considered achieving the first deliverable and would receive the interim reward. The company can opt not to have an interim deliverable, and the incentives would remain as described for ‘final reward’ only and would be awarded on completion of the full PIP requirements. However, rewards should be available for medicinal products, which are not going to be advanced in adults but are beneficial in children.

### 4.2 Proposal 2: incentivise paediatric‐specific drug development

Whilst proposal 1 aims to proportionately reward accelerated paediatric cancer drug development for medicines, which are generally following a development pathway for an adult indication, incentives are needed that motivate and reward investment in paediatric‐specific cancer drug development, uncoupled from adult cancer indications. We propose that the Orphan Regulation can be modified to incentivise paediatric‐specific drug development. Currently, the reward obtained for orphan‐designated medicines is a late reward, which consists of an extended 10 years of market exclusivity. This can once again discourage companies from the vast investment needed to fully develop a new medicine. We propose the introduction of a new ‘early reward’ (Figure 3) that would be granted right after a first marketing authorization for a paediatric cancer indication. This reward could consist of tax reductions, transferable vouchers or other measures that warrant an immediate economic gain, as opposed to the classic late reward. This late reward would, however, remain as it is now.

Potential rewards for developing a drug for a paediatric cancer‐specific indication include accelerated reviews, which are already carried out in Europe within PRIME.^34^ PRIME (PRIority Medicines) is a programme launched by the EMA in 2016 to enhance support for the development of medicines that target an unmet medical need.^34^ This voluntary scheme is based on enhanced interaction and early dialogue with developers of promising medicines, to optimize development plans and speed up evaluation so these medicines can reach patients earlier.^34^ This is achieved through scientific advice and accelerated assessment of medicines applications. The EMA recently published its 5‐year evaluation of PRIME, showing the feasibility and potential benefit of accelerated reviews.^35^ Between 2016 and 2021, 95 requests were granted, with 18 medicines eventually receiving marketing authorization. Out of these 18, seven were oncology drugs. Importantly, the average evaluation time for PRIME medicines was reduced by 6.7 months compared to non‐PRIME medicines.

The review process for marketing authorization applications within EMA differs from that of the FDA; therefore, the concept of a Creating Hope‐like PRV would be difficult to implement in the EU, but nevertheless the concept of a transferable voucher is conceivable. Whatever the approach, the level of this new reward needs to be sufficiently attractive to motivate industry to develop a drug for a potentially non‐profitable market. One approach could be a transferable voucher for the 6‐month extension of the SPC of another drug. This would be a substantial change and would need careful evaluation of the potential socio‐economic impact. The parameters of the drug to which the transferable voucher could be applied need to be carefully defined; for example, the transfer could be restricted only to drugs with a paediatric indication and/or applied only to other compounds in the drug development pipeline and not to products with an existing marketing authorization.

The ability to sell the transferable reward to another company would particularly benefit small companies without an extensive drug development portfolio to which the reward could be applied. This proposal would provide a substantial increase in the incentive to drive specific research and development programmes for the rare paediatric conditions, including paediatric and adolescent cancers that would otherwise not attract investment. In this context, small biotechnology companies can play an important role in the development of drugs for cancers that only occur in children. At ACCELERATE's 2021 annual conference, there was a dedicated session to specifically address the needs of these companies. Generally defined as smaller companies with a primary focus in research and development, biotechnology (biotech) companies do not have the resources of larger pharmaceutical companies. They can be single‐asset companies whose existence depends upon the success of its individual product or platform. As such, biotech companies are less likely to engage early in paediatric drug development, unless that is their sole purpose, or their drug has been specifically designed to do so. Given that the cost of clinical development for an individual programme is in the hundreds of millions of euros, biotech companies often do not have the funds to spend on more than one development programme at a time. As a result, the current incentives are suboptimal for these companies and mainly benefit large pharmaceutical companies. However, smaller biotechnology companies are arguably major drivers of early innovation and have the potential to provide novel drugs to children with cancer, but because of their financial structure, cannot afford to wait for late rewards.^7^ This would align with both our proposals (1 and 2) to change to a segmented reward approach, in which early rewards are offered as part of the PMR and of the Orphan Regulation. Providing rewards during the development of a drug, or post‐marketing, are of no benefit to a biotech company that does not survive to market, because of its lack of resources or early clinical failures, and such companies cannot be expected to deliver paediatric programmes for each asset as a result. Rather, incentives need to be staged and milestone‐driven, reviewed at each step of the development process and with reviews available at each step, instead of at the end.

In addition to moving the timelines of incentives, we propose that novel incentives should be introduced, for example, tax incentives for early investors. Each paediatric indication study should lead to its own incentive/reward (SPC extensions, tax incentives, accelerated reviews). Furthermore, incentives should be transferrable, following the PRV model.

In conclusion, incentives should be implemented earlier rather than later in the drug development process, and be staged, milestone‐driven, novel, proportional to work completed at each phase and transferrable.

## 5 CONCLUSIONS

Drug development for childhood cancers is limited by the imbalance between the resources needed to deliver a full paediatric cancer programme and the potential market reward for doing so successfully. Furthermore, because the profits are coupled to a more lucrative adult drug development market, product innovation unique to paediatric cancers is rarely undertaken. At first glance, incentives to drive industry to invest in drug development for rare paediatric diseases, like childhood cancer, appear to be in place in Europe (Regulation on Orphan Medicinal Products, PMR) and in the US (RACE, Creating Hope Act), but they have not been as effective as was anticipated. The European Pharmaceutical legislation is currently under revision, and we hope that our proposals (Box 1) can be incorporated into the upcoming modifications. We believe the changes in the timing (segmented reward approach) and type (transferable exclusivity voucher) would be of significant benefit to children and adolescents with cancer as well as to other life‐threatening diseases with unmet medical needs. Of note, the segmented reward approach would not increase the overall financial incentives to pharma but is a mechanism to drive more rapid implementation of the goals of the regulation for the benefit of children with life‐threatening diseases.

BOX 1 **Recommendations for the revision of the Paediatric Medicine Regulation (PMR) and the Regulation on Orphan Medicinal Products**

To change the timing and nature of the rewards, which would both drive earlier initiation of paediatric studies and provide incentives for drug development specifically for children.

To modify the PMR to ensure mechanism‐of‐action driven, mandatory paediatric investigation plans

Incentives should be reorganized to a stepwise and incremental approach. Interim and final deliverables should be defined within a PIP, each attracting a reward on completion. An optional interim deliverable would require production of paediatric data that inform the go/no‐go decisions on whether to take a drug forward to paediatric efficacy trials.

To promote paediatric‐specific cancer drug development with the introduction of early rewards in the frame of the Orphan Medicinal Products regulation, with a variant on the US Creating Hope Act and its priority review vouchers.

## AUTHOR CONTRIBUTIONS

**Teresa de Rojas:** Conceptualization (lead); data curation (lead); formal analysis (lead); investigation (lead); methodology (lead); supervision (lead); validation (lead); visualization (lead); writing – original draft (lead); writing – review and editing (lead). **Pamela Kearns:** Conceptualization (lead); data curation (equal); formal analysis (equal); investigation (equal); methodology (equal); supervision (lead); validation (equal); visualization (supporting); writing – original draft (lead); writing – review and editing (equal). **Patricia Blanc:** Conceptualization (equal); data curation (supporting); investigation (supporting); methodology (supporting); supervision (equal); validation (supporting); visualization (supporting); writing – original draft (equal); writing – review and editing (equal). **Jeffrey Skolnik:** Conceptualization (supporting); data curation (supporting); investigation (supporting); supervision (equal); validation (equal); visualization (supporting); writing – original draft (supporting); writing – review and editing (equal). **Elizabeth Fox:** Conceptualization (supporting); investigation (supporting); supervision (equal); validation (equal); visualization (supporting); writing – original draft (supporting); writing – review and editing (equal). **Leona Knox:** Conceptualization (supporting); investigation (supporting); methodology (supporting); supervision (equal); validation (equal); visualization (supporting); writing – original draft (supporting); writing – review and editing (equal). **Raphael Rousseau:** Conceptualization (supporting); investigation (supporting); supervision (equal); validation (supporting); visualization (supporting); writing – original draft (supporting); writing – review and editing (supporting). **François Doz:** Conceptualization (supporting); investigation (supporting); supervision (equal); validation (supporting); visualization (supporting); writing – original draft (supporting); writing – review and editing (equal). **Nick Bird:** Conceptualization (equal); formal analysis (supporting); investigation (equal); supervision (equal); validation (equal); visualization (supporting); writing – original draft (supporting); writing – review and editing (equal). **Andrew Pearson:** Conceptualization (lead); data curation (equal); formal analysis (equal); investigation (equal); methodology (equal); supervision (lead); validation (lead); visualization (equal); writing – original draft (supporting); writing – review and editing (lead). **Gilles Vassal:** Conceptualization (lead); data curation (equal); formal analysis (equal); investigation (lead); methodology (equal); supervision (lead); validation (lead); visualization (equal); writing – original draft (supporting); writing – review and editing (lead).

## FUNDING INFORMATION

Supported by the Andrew McDonough B+ Foundation.

## CONFLICT OF INTEREST

ADJP has consulted for Lilly, Norgine and Developmental Therapeutics Consortium Limited and been an advisor for Amgen. FD has participated in advisory boards for Bayer, BMS, Roche, Celgene, LOXO Oncology, Servier and Tesaro; he has been rewarded for consultancy services by Roche and Servier; he has worked in scientific partnership with Onxeo and Synth‐Innove (all these payments were received in a research account, not a personal account). FD has also been refunded travel expenses by Bayer, BMS and Roche. JS is an employee of Inovio Pharmaceuticals. The rest of the authors have no conflicts of interest to declare.

## ETHICAL APPROVAL

Ethical approval was not sought from an institutional review board nor ethics committee as it is not needed/applicable for this kind of study (no inclusion of human subjects).

## Supporting information


Appendix S1.

## Data Availability

All data collected for the study are publicly available and properly cited in the References.
