# Supplementary material for: Changing incentives to ACCELERATE drug development for paediatric cancer
Source: Cancer Med. 2023 Jan 16;12(7):8825–37. doi: 10.1002/cam4.5627 (PMC10134303; doi:10.1002/cam4.5627)
Supplement: Supplementary file 1 — Appendix S1. [file CAM4-12-8825-s001.docx]

**Supplementary Material:**

**CURRENT FRAMEWORK OF INCENTIVES FOR PAEDIATRIC DRUG DEVELOPMENT**

***The Paediatric Medicines Regulation (PMR)***

The PMR includes a series of obligations and incentives to meet objectives as set out in EC No 1901/2006^1^. The obligatory element requires that companies, when developing a medicinal product for a marketing authorisation (MA), also consider the product’s application in the paediatric age group and submit a Paediatric Investigation Plan (PIP). A PIP should include a description of the studies to be undertaken in children across all applicable age groups from birth to 17 years and any drug formulation adaptations necessary for its use in children, e.g., development of a liquid formulation. The PIP should be submitted for European Medicines Agency (EMA) agreement after completion of adult pharmacokinetic studies, which coincide with the end of phase 1 trials in oncology. The PIP should define the timing of studies in children compared to adults, as well as the measures proposed to assess quality, safety, and efficacy in children.

There are circumstances where the obligations can be waived or deferred. A waiver is possible if the condition for which the product is intended does not occur in the paediatric age group, e.g., lung cancer. In oncology, this has proved extremely controversial because it is known that if the cancer indication for which the MA is being sought does not occur in children, the mechanism by which the drug acts may still be relevant to other cancers occurring in the paediatric age group. There is evidence that the granting of such waivers has prevented children with cancer from having access to potentially active new drugs^2, 3^. The EMA has tried to address this concern in a revision of the class waivers list^4^. However, it remains the case, as specified in the PMR, that unless the intended condition has relevance in a paediatric age group, a company cannot be obliged to submit a PIP. This means that even if the drug has potential relevance in paediatric cancer, a PIP cannot be mandated, and paediatric cancer drug development may not occur^5^.

Within the PMR, the financial incentive for a medicinal product protected by a Supplementary Protection Certificate (SPC) (or a patent qualifying for such a certificate) is the entitlement to a six-month market exclusivity extension if the PIP is completed as agreed with the EMA. This includes updating the labelling of the product to include the paediatric data generated in accordance with the PIP and its submission to the regulatory authority. Also, the applicant must apply for the extension to *each* National Patent Office in the countries where the product is eligible for the extension, at least two years before the expiry of the SPC. Of note, for medicinal products with orphan drug designation (see below), there is an additional 2-year orphan market exclusivity extension (i.e., the total orphan market exclusivity increases to 12 years) if the same conditions are met (except for the requirement to apply two years in advance of the SPC expiry). As such, it has been practically challenging for pharmaceutical companies who wish to meet the needs of children, to do so within the limitations of the SPC-granting mechanisms of the EU.

***Orphan Drug Designation***

The EU does have a mechanism to promote the development of medicines for rare diseases through a specific legal instrument and incentive scheme known as ‘Orphan Drug Designation’. To qualify for Orphan Drug Designation, a drug must be intended to treat, prevent, or diagnose a disease that is life-threatening or chronically debilitating. The disease prevalence must be less than 5 in 10,000 or the product’s market must be unlikely to generate sufficient returns to justify the investment needed for its development, and there must be a significant benefit to patients from the new treatment or no satisfactory method of treatment in the EU. The incentives include a reduction in fees for MA applications and, if authorised, a 10-year orphan market exclusivity for the product in the specific orphan designated indication.

The Orphan Drug Designation route is used frequently in the field of oncology but very rarely for childhood cancers^6^. It is apparent that even for a rare condition occurring both in children and adolescents, the start of the paediatric development is delayed or never occurs. Moreover, for malignancies occurring only in children, the Orphan Drug Designation route is not used by the pharmaceutical industry.

***The US Creating Hope Act: the rare paediatric disease Priority Review Voucher***

The Creating Hope Act in the United States (US) followed a parent-led initiative to address the paucity of drugs developed for paediatric cancers^7, 8^. Following an intensive campaign by the parent-led charity ‘Kids v Cancer’, political lobbyists, the medical community and industry, the Act, based upon prior legislation for rare tropical diseases, was passed in June 2012 and re-authorized in 2020^8^ and led to the use of Priority Review Vouchers to incentivise paediatric drug development in the US. If a company obtains market approval for a drug for a life-threatening/severe paediatric indication (including cancer), it is awarded a Priority Review Voucher that reduces the US Food and Drug Agency (FDA) review time from ten months to six months, gaining four months on potential marketing approval and market access. Importantly, the Voucher is transferable; meaning the benefit can be used for a different drug and indication (with a broader marketing potential) and can be sold to another company. In the US, this priority review process can be extremely beneficial for companies. It means they may be able to market their product more quickly and begin recouping their research and development costs sooner, as well as taking a stronger position against future competition. For patients with rare diseases, the incentive to develop new therapies for that indication is de-coupled from the drug’s direct market value.

***The RACE Act***

The Research to Accelerate Cures and Equity (RACE) for Children Act was passed by the US Congress in 2017 and took effect in 2020^9^. RACE is meant to amend the previous Pediatric Research Equity Act (PREA), enacted in 2003. PREA was designed to give the US FDA the authority to require paediatric studies in certain drugs and biological products and requires sponsors to create Pediatric Investigational Plans (PSP) to define their paediatric drug development strategy. PREA also requires the use or creation of age-appropriate formulations for each age group under investigation. PREA requires sponsors to submit a PSP after the (adult) End-of-Phase 2 meeting, which implies no requirement to start paediatric drug development until required, late in the development process. In addition, PREA originally excluded cancer drugs that had obtained Orphan Drug Designation. RACE for Children Act rectified this exclusion by requiring sponsors of an approved medication whose target may be relevant to the treatment of a paediatric cancer to complete a paediatric assessment as to whether the drug (1) may be used for a substantial number of paediatric cancer patients, or (2) there is reason to believe that the medication would have a meaningful therapeutic benefit over existing therapies for paediatric cancer patients. In either case, the drug would then meet PREA requirements, and require a PSP when the drug’s target is relevant to paediatric cancer. This functionally re-introduced oncology drugs into PREA. FDA maintains an active list of drugs that are relevant to PREA/RACE^10^. However, this still pins paediatric new drug development on adult cancer drug development, rather than considering the merits of paediatric drug development on its own and fails to incentivize paediatric cancer drug development as a result.

**REFERENCES**

**1**. The European Parliament and The Council of the European Union: Regulation No 1901/2006 of the European Parliament and of the Council on Medicinal Products for Paediatric Use and Amending Regulation. Off J Eur Union , 2006

**2**. Vassal G, Zwaan CM, Ashley D, et al: New drugs for children and adolescents with cancer: The need for novel development pathways. Lancet Oncol 14, 2013

**3**. Pearson ADJ, Herold R, Rousseau R, et al: Implementation of mechanism of action biology-driven early drug development for children with cancer [Internet]. Eur J Cancer 62:124–131, 2016[cited 2021 Dec 1] Available from: https://pubmed.ncbi.nlm.nih.gov/27258969/

**4**. European Medicines Agency: European Medicines Agency decision CW/0001/2015 of 23 July 2015 on class waivers, in accordance with Regulation (EC) No 1901/2006 of the European Parliament and of the Council [Internet], 2015[cited 2022 Sep 7] Available from: www.ema.europa.eu/contact

**5**. Vassal G, Blanc P, Pearson A: Need for change in implementation of paediatric regulation [Internet]. Lancet Oncol 14:1156–1157, 2013[cited 2022 Sep 7] Available from: https://pubmed.ncbi.nlm.nih.gov/24176557/

**6**. Vassal G, Kearns P, Blanc P, et al: Orphan Drug Regulation: A missed opportunity for children and adolescents with cancer [Internet]. Eur J Cancer 84:149–158, 2017Available from: http://dx.doi.org/10.1016/j.ejca.2017.07.021

**7**. Kesselheim AS, Maggs LR, Sarpatwari A: Experience With the Priority Review Voucher Program for Drug Development [Internet]. JAMA 314:1687–1688, 2015[cited 2022 May 2] Available from: https://pubmed.ncbi.nlm.nih.gov/26414802/

**8**. Library of Congress: H.R.4439 - 116th Congress (2019-2020): Creating Hope Reauthorization Act [Internet][cited 2022 Sep 7] Available from: https://www.congress.gov/bill/116th-congress/house-bill/4439

**9**. McCaul M: H.R.1231 - 115th Congress (2017-2018): RACE for Children Act [Internet], 2017[cited 2018 Sep 8] Available from: https://www.congress.gov/bill/115th-congress/house-bill/1231

**10**. Food And Drug Administration: Relevant Pediatric Molecular Target List with references [Internet][cited 2022 Sep 7] Available from: https://www.fda.gov/media/120332
